# Housing conditions modify seasonal changes in basal metabolism and body mass of the Siberian hamster, *Phodopus sungorus*

**DOI:** 10.1007/s00360-022-01434-9

**Published:** 2022-03-29

**Authors:** Małgorzata Jefimow, Anna S. Przybylska-Piech

**Affiliations:** 1 Department of Animal Physiology and Neurobiology, Faculty of Biological and Veterinary Sciences, Nicolaus Copernicus University, ul. Lwowska 1, 87‑100 Toruń, Poland; 2 Department of Vertebrate Zoology and Ecology, Faculty of Biological and Veterinary Sciences, Nicolaus Copernicus University, ul. Lwowska 1, 87‑100 Toruń, Poland

**Keywords:** Wheel-running activity, Animal welfare, Siberian hamster, Seasonal changes, Basal metabolic rate, Body mass, Nesting material, Pair housing

## Abstract

Proper housing conditions are important aspects of animal welfare. Animals housed in enriched environments show less stereotypic behaviours than animals kept in barren cages. However, different types of cage enrichment may affect the results of experimental studies and hinder comparative analyses of animal physiology and behaviour. We investigated whether access to a running wheel, availability of nesting material, and pair housing affect basal metabolic rate (BMR) of Siberian hamsters (*Phodopus sungorus*) under various acclimation conditions. We used 70 adult hamsters (35 males and 35 females) divided into five groups housed under different cage conditions. All individuals experienced the same acclimation procedure: first a winter (L8:D16) then a summer (L16:D8) photoperiod, at air temperatures of first 20 °C then 7 °C under both photoperiods. We found that nesting material and pair housing did not affect hamster BMR, while access to a running wheel increased BMR and body mass regardless of photoperiod and ambient temperature. Thus, we suggest that cage enrichment should be applied with caution, especially in studies on energetics or thermoregulation, particularly in seasonal animals.

## Introduction

Cage enrichment like access to tunnels, running wheels, balance beams, shelters, climbing structures, or nesting material, and in many species also group housing, improve the welfare of captive animals by providing external stimuli or social contact, reinforcing activity, and preventing monotony. This is especially pertinent to laboratory animals. Despite many advantages of cage enrichment, there is a concern that non-standard equipment in a cage may bias experimental results and hinder comparative studies (Bailoo et al. 2018). Indeed, energy expenditure of laboratory animals depends on the activity in the running wheel (Goodrick 1980) and availability of nesting material (Van de Weerd et al. 1997). It can also vary with the number of individuals housed per cage (Nuñez-Villegas et al. 2014).

Although access to running wheel is beneficial for animal wellbeing (Goodrick 1980; Lambert and Noakes 1990; van Praag et al. 1999), it may increase energy expenditure and modify seasonal changes in physiology (Borer et al. 1983; Scherbarth et al. 2007). In this study, we asked whether the effects of voluntary activity on basal metabolic rate (BMR) of a seasonal mammal change with photoperiod and ambient temperature (*T*_a_). Wheel-running activity may affect body mass and body composition (Allen et al. 2001; Kemi et al. 2002; Houle-Leroy et al. 2003; Waters et al. 2004; Swallow et al. 2005; Scherbarth et al. 2007; Petri et al. 2010; Soffe et al. 2016; Kelly et al. 2017), and it may stimulate growth or increase in bone density (Scherbarth et al. 2007; 2008). Likewise, seasonal phenomena, like winter decrease in body mass (*m*_b_) (Scherbarth et al. 2007, 2008; Petri et al. 2014), winter gonadal regression (Gibbs and Petterborg 1986; Scherbarth et al. 2007), daily torpor expression (Thomas et al. 1993; Scherbarth et al. 2007), and hibernation torpor (Pengelley and Fisher 1966) are also prevented or delayed by voluntary exercise in running wheel. Locomotor activity itself also depends on season (Kenagy 1973; O'Farrell 1974; Conner 1983; Ebensperger and Hurtado 2005; Paise and Vieira 2006), and voluntary exercise affects neuroendocrine function, hormone secretion and somatic growth (Borer et al. 1983). Finally, running wheel activity induces an increase in energy expenditure and generates heat that can be used for thermoregulation (Wunder 1970; Refinetti 1994; Weinert and Waterhouse 1998; Chappell et al. 2004; Vaanholt et al. 2007; Weinert et al. 2018). It was also found that mice selected for high nest-building behaviour were less active in running wheel than controls or low-nest builders (Bult et al. 1993).

The second goal of our study was to estimate the effects of thermal microenvironment in a cage on BMR. We quantified the effects of additional nesting material and housing in pairs on the seasonal changes in BMR. Shape and size of animal’s nest depends on the species (Yunes et al. 1991), strain (Lynch and Hegmann 1972), body mass (Lynch and Roberts 1984), ambient temperature (Gaskill et al. 2011), and season (Puchalski et al. 1988; Przybylska et al. 2019b). The quality and quantity of nesting material can also affect the energy expenditure of laboratory animals (Van de Weerd et al. 1997). The presence of nest in a cage facilitates thermoregulation and increases thermal comfort of an individual, particularly as standard laboratory *T*_a_ is usually below the thermoneutral zone of most small laboratory rodents (Gordon 1990, 1993, 2012; Jefimow et al. 2003). For example, laboratory mice (C57BL and BALB strains) with access to nesting material had higher *m*_b_ and consumed less food than mice from barren cages, suggesting reduced energy expenditure for thermoregulation (Van de Weerd et al. 1997). In Siberian hamsters, seasonally intensified nest-building behaviour likely reflects an intrinsic drive to build more insulated nests during winter (Puchalski et al. 1988; Przybylska et al. 2019b). Because group housing may reduce energy expenditure by reducing surface-to-volume ratio of grouped animals (Contreras 1984), we also studied the effect of pair-housing on seasonal changes in BMR. Although Siberian hamster is rather solitary than social (Wynne-Edwards 2003), it can be housed in groups of the same-sex littermates (Jefimow et al. 2011).

As a model we used a highly photosensitive species, the Siberian hamster (*Phodopus sungorus*). Many studies examined different aspects of energy expenditure, including metabolic rate, body temperature, nonshivering thermogenesis, daily torpor, seasonal changes in *m*_b_, and activity rhythms in this species (Figala et al. 1973; Hoffmann 1973; Heldmaier 1975b; Steinlechner et al. 1983; Heldmaier et al. 1985, 1989; Heldmaier 1989; Weiner and Heldmaier 1987; Puchalski and Lynch 1988; Jefimow et al. 2004). The hamsters respond to short photoperiod by adjusting several traits that constitute its winter phenotype. Namely, they decrease *m*_b_, molt to a white fur, regress gonads and use daily torpor (Figala et al. 1973; Hoffmann 1973; Heldmaier and Steinlechner 1981a, b; Lynch and Puchalski 1986; Ruf and Heldmaier 1992; Ruf et al. 1993; Przybylska-Piech et al. 2021). Boratyński et al. (2016) also found that Siberian hamsters acclimated to winter-like conditions had lower whole animal BMR than summer-acclimated ones.

To answer our questions we measured BMR, voluntary activity in running wheels, and changes in body mass of Siberian hamsters acclimated to winter-like, short, and summer-like, long photoperiods at ambient temperatures of both 20 and 7 °C. We predicted that continuous access to a running wheel would induce an increase in whole animal BMR independent of season. Further, we predicted that BMR would be lower in animals that have access to nesting material and that BMR will be lower in animals acclimated to winter-like than to summer-like conditions. Finally, we expected that housing in pairs would result in lower BMR compared to hamsters housed solitarily.

## Material and methods

### Ethical note

All experiments received ethical approval from the Local Committee for Ethics in Animal Research in Bydgoszcz, Poland (decision no. 5/2020).

### Animals and housing

Siberian hamsters used in these experiments were from our breeding colony kept at the Faculty of Biological and Veterinary Sciences at the Nicolaus Copernicus University in Toruń. All animals descended from hamsters obtained from the University of Halle-Wittenberg and Philipps University of Marburg, Germany. We used 70 adult hamsters (35 males and 35 females) born under summer-like conditions (16L:8D, *T*_a_ = 20 ± 2 °C). After weaning at 18–19 day of age, all hamsters were initially housed in same sex pairs. At the age of ~ 3 months, hamsters were exposed to a winter-like photoperiod (8L:16D, *T*_a_ = 20 ± 2 °C) for 4 months. During this initial acclimation, animals were housed either individually or in pairs in standard laboratory cages (220 × 165 × 140 mm high) with wood shavings and paper tubes for bedding and nesting material. Food (standard rodent diet; Labofeed B, Morawski, Kcynia, Poland) and water were available ad libitum. Paired animals were kept together throughout entire experiment and constituted the first experimental group that included 3 male–male and 4 female–female pairs (Group P: Pair-housed animals). The other hamsters were housed singly, and divided into four groups maintained in experimental cages that differed in size, availability of a running wheel, and nesting material (Table 1). Each group of solitary hamsters consisted of 14 individuals (7 males and 7 females). Hamsters in Group S (Single animals) were housed singly in standard laboratory cages with wood shavings as bedding material (barren cage). Animals from Group SN (Single animals with nesting material) were supplemented with nesting material (paper tube and paper towel). Hamsters from Group W (Wheel in a cage) were housed in larger cages with running wheel and bedding material (320 × 165 × 140 mm high, wheel circumference = 76.65 cm) and individuals from Group WN (Wheel and Nesting material) were kept in cages with a running wheel and nesting material (paper tube and paper towel).

**Table 1:** Housing conditions of five experimental groups. Only during the initial acclimation periods hamsters were housed in cages with the same enrichment (small with nesting material)

Group	*N*	Number of individuals per cage	Cage size	Wood shavings	Nesting material	Running wheel
P	3 pairs ♂–♂ 4 pairs ♀–♀	2	Small	Yes	Yes	No
S	7♀; 7♂	1	Small	Yes	No	No
SN	7♀; 7♂	1	Small	Yes	Yes	No
W	7♀; 7♂	1	Large	Yes	No	Yes
WN	7♀; 7♂	1	Large	Yes	Yes	Yes

*P* pair-housed animals, *S* single animals, *SN* single animals with nesting material, *W* animals housed in cages with running wheel, *WN* animals housed in cages with running wheel and nesting material

**Fig. 1:**
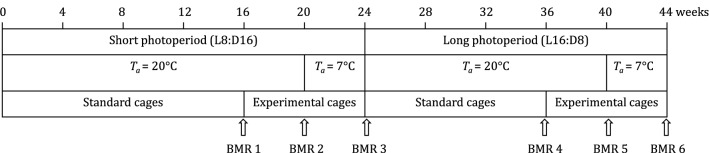
Timeline of acclimation to different photoperiods and ambient temperatures (*T*_a_) and measurements of basal metabolic rate (BMR) in Siberian hamsters

BMR of each individual was measured before dividing hamsters to cages varying in captive conditions (BMR1), after 4 weeks of cage treatments at *T*_a_ = 20 ± 2 °C (BMR2), and after a further four weeks at *T*_a_ = 7 ± 2 °C (BMR3). After 24 weeks under winter-like conditions, photoperiod and *T*_a_ were changed to summer-like conditions (16L:8D, *T*_a_ = 20 ± 2 °C) and hamsters were transferred to standard laboratory cages as described above. After 12 weeks of initial acclimation to the summer conditions, we measured BMR three times, in the same order and manner as during the winter photoperiod (BMR 4, 5 and 6; Fig. 1).

Hamsters were weighed every week or every 2 weeks to an accuracy of ± 0.1 g with an electronic balance (SPU402, Ohaus, U.S.A.) to monitor changes in *m*_b_. Body mass was also measured before and after each BMR measurement. Two animals (one from group W and one from group WN) died during acclimation from unknown reasons, and thus our final sample size is 68 individuals.

### Basal metabolic rate

Basal metabolic rate (BMR) was measured by indirect calorimetry using an open flow respirometry system at *T*_*a*_ = 28.5 °C, which is within the thermoneutral zone of Siberian hamsters (Gutowski et al. 2011). Measurements were done during the animals' rest phase, and lasted for approximately 7.5 h. We used two parallel respirometry systems, allowing us to measure gas exchange in 14 hamsters simultaneously (7 per system per day). Air was sequentially sampled from each animal chamber at 5-min intervals, with reference air being sampled for 4 min at least every 15 min, and this cycle was repeated throughout measurements. Thus the gas exchange of each hamster was measured every 44 min throughout the day. Air was pulled from outside the building using an air pump (DOA-P501-BN, Gast Manufacturing INC., Michigan, USA), then it was dried using silica gel and was continuously pushed through 0.85 L airtight metabolic chambers constructed of translucent polypropylene containers (HPL 808, Lock & Lock, Hana Cobi, South Korea) at a constant flow rate of ~ 430 mL min^−1^. All chambers were placed in a temperature-controlled cabinet (ST-1200, Pol-Eko-Aparatura, Wodzisław Śląski, Poland). Airflow was regulated upstream of the respirometry chambers using precise needle valves. Gases leaving the respirometry chambers were selected sequentially by a computer-controlled multiplexer (MUX, Sable Systems Int., Las Vegas, NV, U.S.A.) and the flow rate of each chamber was measured downstream using a mass flow meter (FlowBar-8, Sable Systems Int.; calibrated against a volumetric calibrator (Defender 530 + , Mesa Laboratories, Inc., Butler, NJ, USA). A multiplexer received air from all 14 chambers and selected two separate airstreams leading to different sets of gas analysers. After flow measurement, air from each gas stream was subsampled at a rate of ~ 100 mL min^−1^ and water vapour pressure of the subsampled air was measured with a water vapour analyser (RH-300, Sable Systems Int.). Air was then dried in a column of magnesium perchlorate (Sigma-Aldrich, U.S.A.), and subsequently fractional concentrations of CO_2_ (*F*CO_2_) and O_2_ (*F*O_2_) were measured using a FoxBox-C integrated CO_2_ and O_2_ analyser, or with a FC-10a O_2_ analyser (Sable Systems Int.) and CA10 CO_2_ analyser (Sable Systems Int.). Water vapor and CO_2_ analyzers were calibrated prior to each series of measurements against N_2_ (zero) and known concentrations of H_2_O or CO_2_ in N_2_. O_2_ analysers were spanned daily against dry atmospheric air. All electronic outputs of the respirometry system were sent to a PC via an analogue-to-digital interface (UI2, Sable Systems Int.). Respirometry data were recorded using ExpeData software (Sable Systems Int.) at 0.5 Hz and $\dot{V}{\text{O}}_{2}$ and $\dot{V}{\text{CO}}_{2}$ were calculated using Eqs. 11.7 and 11.8 in Lighton (2008). Metabolic rate (MR, W) was calculated using the oxyjoule equivalent in Lighton et al. (1987):

$$ {\text{MR }}\left( {\text{W}} \right) = \frac{{\dot{V}{\text{O}}_{2} \left( {16 + 5.164 \cdot {\text{RER}}} \right)}}{60}, $$

where $\dot{V}{\text{O}}_{2}$ is the rate of oxygen consumption (ml O_2_/min) and respiratory exchange ratio $(\text{RER)} = \frac{{\dot{V}{\text{CO}}_{2} }}{{\dot{V}{\text{O}}_{2} }}$.

### Locomotor activity

Running wheel activity was recorded using a LabJack U3 programmable AD interaface (LabJack, Lakewood, CO, USA) with a DAQFactory (AzeoTech Inc., Ashland, OR, USA) and routine prepared by Paweł Koteja (unpublished). The program records the binary state (moving or stationary) of the wheel motion sensor in 0.02 s increments, and saves it in an output file as means for successive 10-s intervals. The software did not allow us to count intensity of activity or distance covered, and therefore activity was calculated as the percent of time active by day and by night as well as duration of activity in hours.

### Statistical analysis

#### Body mass

For the analyses of *m*_b_ changes over time we used data that were recorded after each acclimation period, i.e. at the time of BMR measurements. It was done in two separate analyses using linear mixed-effect models (LMM) with type III Sums of Squares. To fit linear mixed models we used package lme4 (Bates et al. 2015) and for post-hoc comparison of estimated marginal means we used package emmeans (Lenth 2020) in R v. 4.0.3 (R core 2020). In the set of analyses, we tested the effect of housing conditions (standard cage or larger cage with a running wheel), nesting material (present or absent), photoperiod (long or short days) or acclimation period (initial acclimation in standard cages, acclimation to experimental cages at 20 °C, and then at 7 °C) on changes in *m*_b_ of single-housed hamsters. In the second analysis we tested the effect of housing in pairs on *m*_b_. We compared *m*_b_ of pair-housed individuals (group P) with *m*_b_ of solitary animals housed in standard cages with nesting material (group SN). To build initial models we used Regression with Empirical Variable Selection approach (Goodenough et al. 2012). This approach consists of creating a series of models that include independent variables and their interactions with the most empirical support. Then, we selected minimum models based on the Akaike information criteria with a correction for small sample size (AICc) calculated using package MuMIn (Bartoń 2020). Animal identity (ID) was included as a random factor in both analyses to control for repeated measurements of individuals. Type of cage and nesting material were retained as fixed factors in final model of the first analysis, and the effect of housing in pairs remained in final model of the second analysis. Therefore, the final model analysing effect of housing conditions on *m*_b_ dynamics in solitary hamsters included type of cage, nesting material, photoperiod, acclimation period, and sex as fixed factors and all possible interactions between type of cage, acclimation period and photoperiod. The final model describing the effect of pair housing included housing in pairs, photoperiod, acclimation period, and sex as fixed factors and the interaction: photoperiod × acclimation period.

#### Basal metabolic rate

We analysed BMR in a similar way as *m*_b_. We did two separate analyses of BMR using LMM (lme4 (Bates et al. 2015)). In the first analysis we tested the effect of housing conditions, photoperiod, and acclimation period on BMR of single-housed hamsters. The second analysis of BMR tested the effect of pair-housing on BMR. In both analyses hamster ID was included as a random factor. Body mass was included and kept as covariate in all tested models. Changes in metabolic rate were analyzed with *m*_b_ as a covariate because this approach allows discriminating between mass-dependent and mass-independent differences in metabolic rate between groups (Packard and Boardman 1988; Tschöp et al. 2012; Fernández-Verdejo et al. 2019; Müller et al. 2021). Type of cage and nesting material were retained as fixed factors in final model of the first analysis, and the effect of housing in pairs was retained in final model of the second analysis. Next to body mass, the final model of the first analysis included type of cage, nesting material, photoperiod, acclimation period, and sex as fixed factors and the interactions of photoperiod × acclimation period, and acclimation period × type of cage. The final model of the second analysis included housing in pairs, photoperiod, acclimation period, and sex as fixed factors and *m*_b_ as covariate. The results of the type III analysis of variance are given in Tables 2 and 3.

**Table 2:** Results of the type III analysis of variance calculated for body mass (*m*_b_) and basal metabolic rate (BMR) of single-housed hamsters showing the effects of housing conditions (standard cage or larger cage with a running wheel), nesting material (present or absent), photoperiod (long or short days) and acclimation period (initial acclimation in standard cages, acclimation to experimental cages at 20 °C, and then at 7 °C)

Single-housed animals			
Trait	Factors	F(df)	*P* value
*m*_b_	Type of cage	23.280 (1, 50)	< 0.001
	Nesting material	0.033 (1,50)	0.856
	Photoperiod	317.012 (1, 260)	< 0.001
	Acclimation period	9.232 (2, 260)	< 0.001
	Sex	20.580 (1, 50)	< 0.001
	Photoperiod × acclimation period	56.067 (2, 260)	< 0.001
	Type of cage × photoperiod	15.959 (1, 260)	< 0.001
	Type of cage × acclimation period	14.434 (2, 260)	< 0.001
	Photoperiod × acclimation period × type of cage	11.269 (2, 260)	< 0.001
BMR	*m*_b_	528.923 (1, 183)	< 0.001
	Type of cage	28.173 (1, 60)	< 0.001
	Nesting material	1.120 (1, 49)	0.278
	Photoperiod	76.932 (1, 311)	< 0.001
	Acclimation period	164.661 (2, 266)	< 0.001
	Sex	6.582 (1, 59)	0.013
	Photoperiod × acclimation period	14.901 (2, 280)	< 0.001
	Type of cage × acclimation period	18.547 (2, 268)	< 0.001

Statistical significance was accepted at α ≤ 0.050

**Table 3:** Results of the type III analysis of variance calculated for body mass (*m*_b_) and basal metabolic rate (BMR) of single vs. paired-housed hamsters showing the effects of housing in pairs (pair-housed individuals or solitary animals housed in standard cages with nesting material), photoperiod (long or short days) and acclimation period (initial acclimation in standard cages, acclimation to experimental cages at 20 °C, and then at 7 °C)

Paired-housed animals			
Trait	Factors	F(df)	*P *value
*m*_b_	Housing in pairs	0.042 (1, 25)	0.839
	Photoperiod	225.629 (1, 135)	< 0.001
	Acclimation period	1.901 (2, 135)	0.152
	Sex	37.784 (1, 25)	< 0.001
	Photoperiod × acclimation period	18.651 (2, 135)	< 0.001
BMR	*m*_b_	284.327 (1, 121)	< 0.001
	Housing in pairs	0.276 (1, 24)	0.604
	Photoperiod	17.415 (1, 161)	< 0.001
	Acclimation period	40.467 (2, 137)	< 0.001
	Sex	5.054 (1, 40)	0.030

Statistical significance was accepted at α ≤ 0.050

#### Wheel running activity

We tested the effect of nesting material, photoperiod, acclimation, and phase of day on percentage of time spent in activity (%) and absolute time (hours) spent in activity in solitary hamsters using LMM (LMM4 (Bates et al. 2015)). We analysed data from daytime and nighttime separately as data points for activity in these periods did not overlap and the analysis for the entire range did not meet the assumptions of linear modelling. In all analyses animal ID was included as random factor. Since we asked about the effect of nesting material on activity, it was retained as fixed factor in all models.

The final model for the percentage of time spent in activity during daytime included nesting material and photoperiod as fixed factors, while the final model for absolute time of daytime activity included only nesting material as fixed factor. Other fixed factors were excluded from analyses because they did not affect wheel running activity and decreased model fit. The final model for relative nighttime activity as the percentage of time included nesting material, photoperiod, acclimation period, and sex as fixed factors, and the interaction of photoperiod and acclimation period. The final model for absolute nighttime activity included the same factors except for sex.

The correlation between activity and BMR was analysed using package stats (R Core 2020) in R v. 4.03. We used Kendall rank correlation coefficient (Kendall's tau) because activity data was not normally distributed. Because whole animal BMR increased with body mass, we used residuals from the relationship between *m*_b_ and BMR and analysed their correlation with time spent in activity (%), and with absolute time (hours) spent in activity both at night and during the day. All results are presented as estimated marginal means ± SE and were compared pairwise using Tukey’s HSD test adjusted for multiple comparisons (Lenth 2020). All estimated marginal means from models describing variability of BMR were adjusted for the variation in *m*_b_. Statistical significance was accepted at α ≤ 0.050.

## Results

### Body mass

**Fig. 2:**
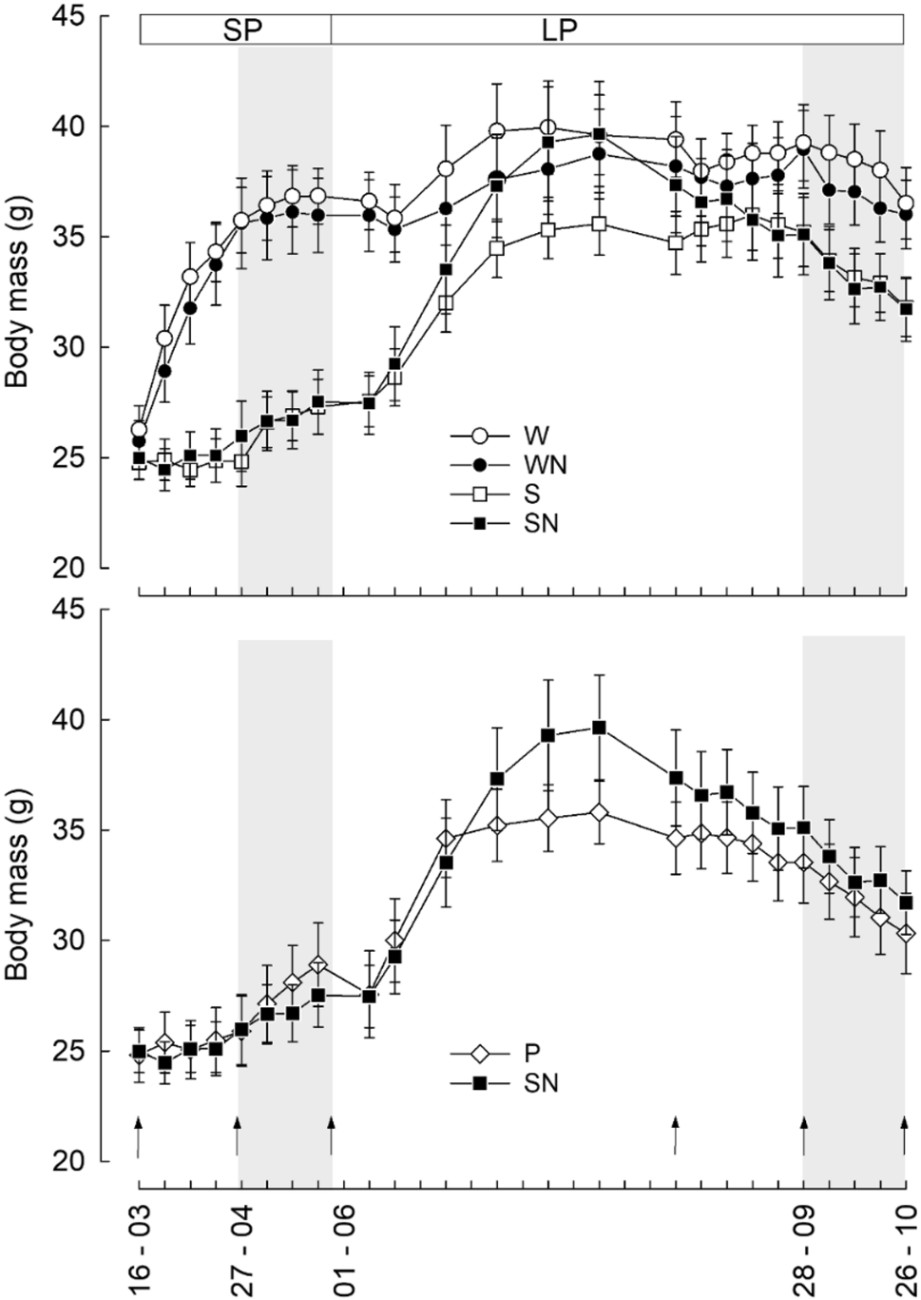
Upper panel: Changes in body mass (mean ± SE, g) over time in Siberian hamsters housed in cages with a running wheel (W), with a running wheel and nesting material (WN), in small barren cages (S), and in small cages with nesting material (SN). Lower panel: Changes in body mass (mean ± SE, g) over time in Siberian hamsters housed in small cages in pairs (P) and in small cages with nesting material (SN). Top bars indicate winter, short photoperiod (SP), and summer, long photoperiod (LP). Ambient temperature (*T*_a_) was set to 20 ± 2 °C except for the periods marked with grey vertical bars, when *T*_a_ = 7 ± 2 °C. Arrows indicate basal metabolic rate (BMR 1–6) measurements

Changes in *m*_b_ varied with type of cage, photoperiod, and acclimation (LMM: photoperiod × acclimation period × type of cage; F(1, 260) = 11.269, *P* < 0.001; Fig. 2). At the beginning of the experiment (initial acclimation in short days), *m*_b_ of hamsters with access to a running wheel (25.6 ± 0.96 g) and without (24.4 ± 0.92 g) did not differ (Tukey’s HSD *P* = 0.386). Later, hamsters with access to a running wheel increased *m*_b_ in short photoperiod to 34.5 ± 0.96 g at 20 °C (Tukey’s HSD *P* < 0.001) and then maintained it stable at 7 °C (35.4 ± 0.96 g; Tukey’s HSD *P* = 0.562). Hamsters housed in standard cages did not change *m*_b_ significantly during acclimation to 20 °C (24.8 ± 0.92 g; Tukey’s HSD *P* = 0.926) but increased it at 7 °C (27.0 ± 0.92 g; Tukey’s HSD *P* = 0.042). As a result, hamsters with access to running wheels were heavier than hamsters housed in standard cages at the end of the short-day exposure (35.4 ± 0.96 g vs. 27.03 ± 0.92 g; Tukey’s HSD *P* < 0.001). During initial acclimation to long days these two groups still differed (38.2 ± 0.96 g vs. 35.3 ± 0.92 g; Tukey’s HSD *P* = 0.027). Then, after 4 weeks of acclimation to different housing conditions under long days and *T*_a_ = 20 °C animals did not change *m*_b_ and individuals with access to running wheels were still heavier than individuals housed in standard cages (37.5 ± 0.96 g and 34.6 ± 0.92 g, respectively; Tukey’s HSD *P* = 0.031). During acclimation to 7 °C under long photoperiod, all hamsters lost *m*_b_, but hamsters with access to wheels lost less *m*_b_ and at the end of experiment were heavier (35.4 ± 0.96 g) than animals without access to a wheel (31.0 ± 0.92 g; Tukey’s HSD *P* = 0.001). Also males were heavier than females (LMM: F(1, 50) = 20.580, *P* < 0.001). We did not record any significant effect of nesting material on hamster *m*_b_ (LMM: F(1, 50) = 0.033, *P* = 0.856).

Single-housed and pair-housed hamsters did not differ in *m*_b_ (LMM: F(1, 25) = 0.042, *P* = 0.839), either under short days (25.9 ± 0.94 g and 25.6 ± 0.94 g, respectively), or under long days (29.7 ± 0.90 g and 29.4 ± 0.90 g, respectively).

### Basal metabolic rate

**Fig. 3:**
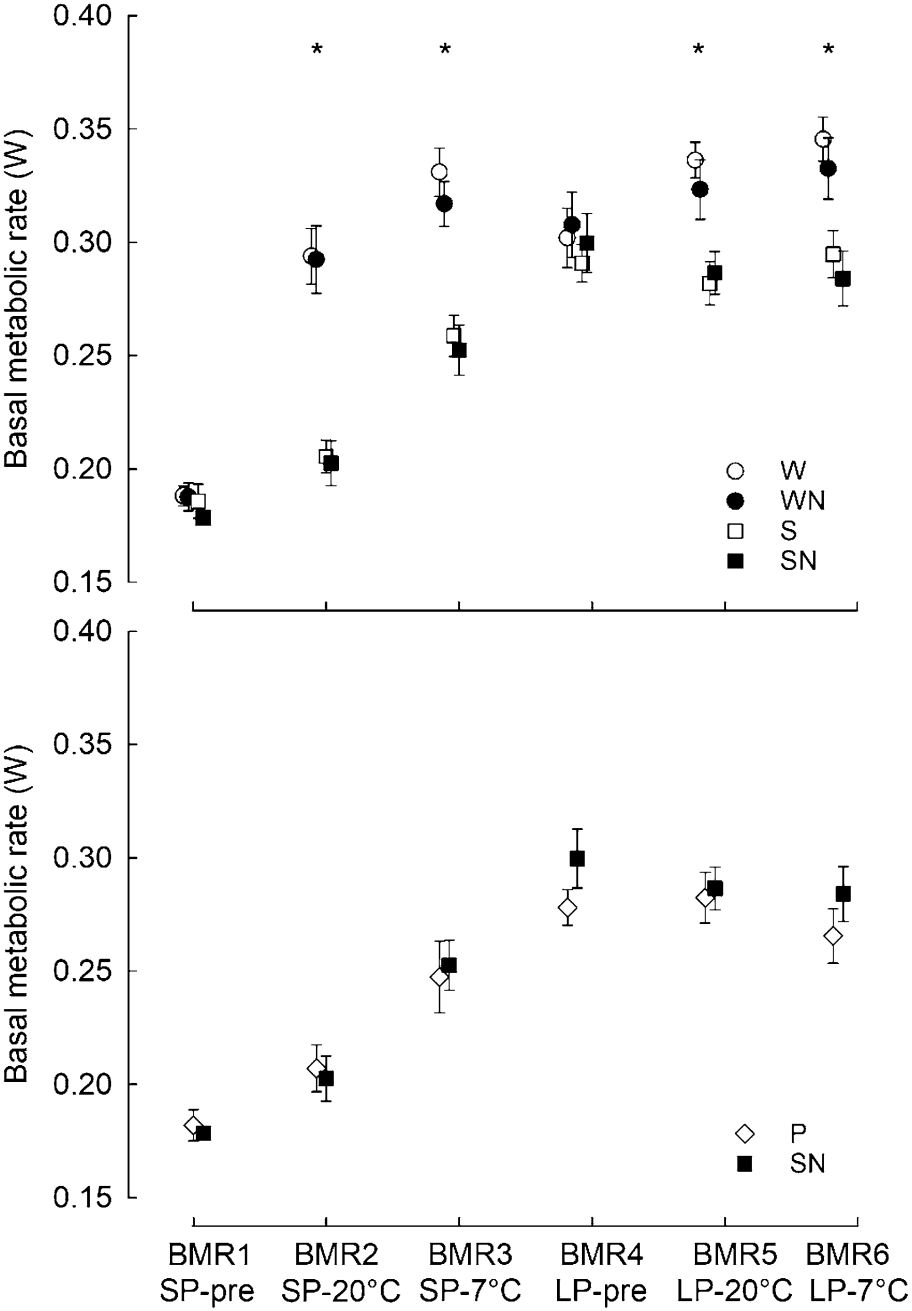
Upper panel: Basal metabolic rate (mean ± SE, W) was measured during initial acclimation to short photoperiod (SP-pre), after 4 weeks under SP and *T*_a_ = 20 ± 2 °C (SP-20 °C), after 4 weeks under SP and *T*_a_ = 7 ± 2 °C (SP-7 °C), after initial acclimation to long photoperiod (LP-pre), after 4 weeks under LP and *T*_a_ = 20 ± 2 °C (LP-20 °C), and after 4 weeks under LP and *T*_a_ = 7 ± 2 °C (LP-7 °C). Siberian hamsters were housed in cages with a running wheel (W), with a running wheel and nesting material (WN), in small barren cages (S), and in small cages with nesting material (SN). Stars indicate significant difference (P < 0.001) in BMR caused by an access to running wheel (within the same time of BMR measurements). Lower panel: Basal metabolic rate (mean ± SE, W) of Siberian hamsters housed in small cages in pairs (P) and in small cages with nesting material (SN)

Basal metabolic rate increased with *m*_b_ (LMM: F(1, 183) = 528.923, *P* < 0.001). Overall, after controlling for *m*_b_ hamsters had higher BMR under long (0.286 ± 0.002 W) than short photoperiod (0.261 ± 0.002 W; LMM: F(1, 312) = 76.932, *P* < 0.001; Fig. 3) and females had higher BMR (0.277 ± 0.002 W) than males (0.270 ± 0.002 W; LMM: F(1, 59) = 6.582, *P* = 0.013). Under both photoperiods, BMR increased throughout acclimation periods (LMM: F(2, 266) = 164.661, *P* < 0.001), but an increase in short days was higher (from 0.226 W to 0.294 W) than in long days (from 0.271 W to 0.306 ± 0.003 W; LMM: photoperiod × acclimation period; F(1, 280) = 14.901, *P* < 0.001).

When controlled for *m*_b_, BMR of hamsters with access to running wheels was higher than BMR of individuals maintained in standard cages (0.281 ± 0.002 W and 0.265 ± 0.002 W, respectively; LMM: F(1, 60) = 28.173, *P* < 0.001; Fig. 3), and this difference was affected by acclimation period (LMM: type of cage × acclimation period; F(1, 268) = 18.547, *P* < 0.001). During the initial acclimation (both in short and in long days), when all individuals were housed under the same conditions, hamsters did not differ in BMR (Tukey’s HSD *P* = 0.373). Then, hamsters with access to running wheels had higher BMR than individuals maintained in standard cages, both at 20 °C (0.287 ± 0.003 W and 0.257 ± 0.003 W, respectively; Tukey’s HSD *P* < 0.001) and after acclimation to 7 °C (0.311 ± 0.003 W and 0.289 ± 0.003 W, respectively; Tukey’s HSD *P* < 0.001). Irrespective of the day length animals that had access to running wheels increased BMR throughout consecutive acclimations (Tukey’s HSD *P* < 0.001), whereas hamsters maintained in standard cages increased their BMR only after acclimation to 7 °C (Tukey’s HSD *P* < 0.001), and did not change BMR between initial acclimation period and acclimation to 20 °C (Tukey’s HSD *P* = 0.231).

The availability of nesting material had no effect on hamster BMR (LMM: F(1, 49) = 1.120, *P* = 0.278). Analysing the effect of housing in pairs, we found the same effect of photoperiod (LMM: F(1, 161) = 17.415, *P* < 0.001), acclimation (LMM: F(2, 137) = 40.467, *P* < 0.001), and body mass (LMM: F(1, 121) = 284.327, *P* < 0.001) as in the first analysis. Housing in pairs had no effect on BMR (LMM: F(1, 24) = 0.276, *P* = 0.604). After controlling for *m*_b_, BMR of single- and pair-housed individuals was 0.248 ± 0.003 W and 0.246 ± 0.003 W, respectively.

### Wheel running activity

**Fig. 4:**
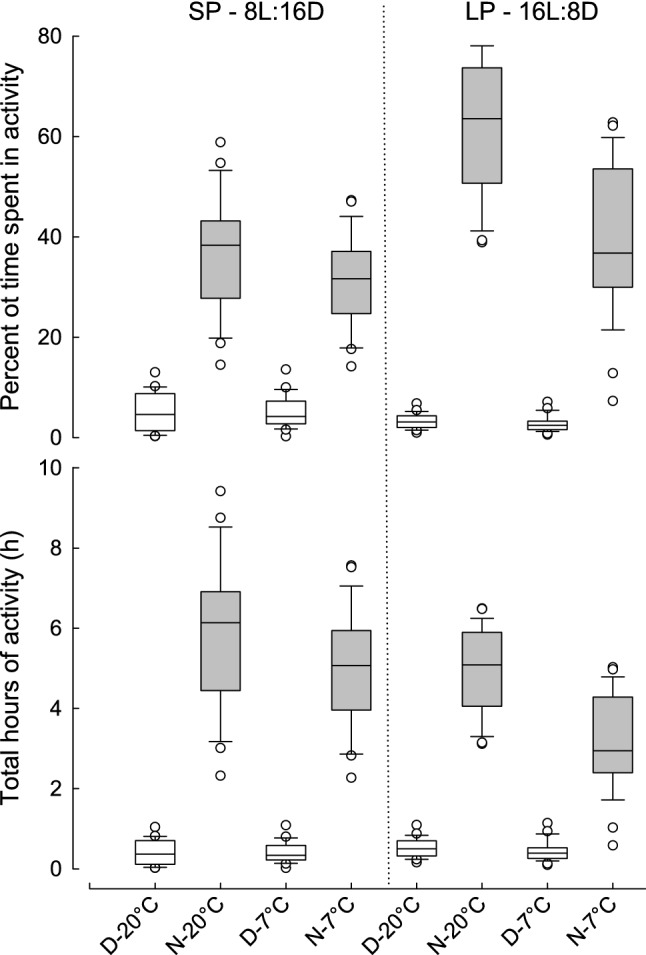
Day (white bars) and night (grey bars) activity of Siberian hamsters at 20 and 7 °C, under short (SP) and long (LP) photoperiods. Data are presented as the percent of time that hamsters spent active (upper panel) and as total hours of activity (bottom panel). Data for hamsters housed in running wheel cages with and without nesting material were pooled as there were no differences between them. Horizontal lines within boxes indicate medians, boxes cover the 25th to 75th percentiles, whiskers indicate the 10th and 90th percentiles, and dots indicate outliers. Statistical significant differences are provided in the Results section

The percentage of time active in the running wheel during the day was higher in short (5.18 ± 0.43%) than in long days (2.98 ± 0.43%; LMM: F(1, 77) = 25.005, *P* < 0.001; Fig. 4). Availability of nesting material affected neither the percentage of time active during the light phase of the day (LMM: F(1, 24) = 0.011, *P* = 0.917) nor the absolute time (hours) spent active per day (LMM: F(1, 24) = 0.011, *P* = 0.917). The percentage of time active during the night was higher in long days (50.7 ± 2.13%) than in short days (33.9 ± 2.13%; LMM: F(1, 75) = 118.700, *P* < 0.001). In contrast, the absolute time active per night was higher in short (5.42 ± 0.24 h) than in long days (4.05 ± 0.24 h; F(1, 75) = 61.649, *P* < 0.001). Daily activity did not correlate with residual BMR when measured as the percentage of time (tau = − 0.060, *P* = 0.360) and as the absolute time spent in activity (tau = 0.027, *P* = 0.686).

The effect of acclimation temperature on nighttime wheel running activity in both percentage of time and as absolute time depended on photoperiod (LMM: photoperiod × acclimation period; F(1, 75) = 27.670, *P* < 0.001 and F(1, 75) = 5.688, *P* = 0.020, respectively). The percentage of time active by night was highest after acclimation to long days at 20 °C (61.7 ± 2.39%), whereas it was similar after acclimation to long days at 7 °C, short days at 20 °C and 7 °C (39.7 ± 2.39%, 36.8 ± 2.39%, and 31.1 ± 2.39%, respectively). Therefore, the difference in nighttime running activity between short and long days was much larger at 20 °C than at 7 °C, and the difference between activity at 7 °C and 20 °C was higher in long than in short days. The absolute time active by night was highest after acclimation to short days and 20 °C (5.88 h ± 0.27 h), and lowest after acclimation to long days and 7 °C (3.17 ± 0.27 h), whereas after acclimation to short days and 7 °C (4.96 ± 0.27 h) it was similar to activity under long photoperiod and 20 °C (4.93 ± 0.27). Therefore, the differences between short and long days were more pronounced at 7 °C than at 20 °C, and the difference between 7 °C and 20 °C was higher under long than under short photoperiod (Fig. 4). Activity during the night was not correlated with residual BMR, either when measured as the percentage of time (tau = 0.057, *P* = 0.390) nor as the absolute time spent in activity (tau = − 0.107, *P* = 0.107).

Availability of nesting material affected neither percentage of time spent in activity during the night (LMM: F(1, 23) = 0.735, *P* = 0.400) nor the absolute time spent active per night (LMM: F(1, 24) = 0.754, *P* = 0.394).

## Discussion

Cage enrichment and group housing ensure animal welfare. However, there is a concern that variability of items provided to the cage may translate to differences in animal physiology, which may bias experimental results, and hamper comparative analyses. We predicted that housing conditions would affect seasonal changes of BMR of small rodent, what could be a source of error in studies involving inter- and intraspecific comparisons. We found that Siberian hamsters housed in cages with running wheels had higher whole animal BMR and higher *m*_b_ than animals housed in standard cages independent of photoperiod and *T*_a_. Conversely, nesting material allowing building the nests, as well as housing in pairs, did not affect BMR and *m*_b_. These results indicate that running wheels should be used with care particularly for studies involving comparisons of physiological traits like metabolic rate.

## Effects of running wheel activity

Hamsters housed in cages with running wheels had higher whole animal BMR than hamsters housed without wheels, and this difference was not explained by changes in *m*_b_, photoperiod or *T*_a_. Raichlen et al. (2010) pointed out that variation in BMR is strongly correlated with variation in muscle mass. This explanation seems plausible because there were no metabolic differences between groups during initial acclimation to winter and summer photoperiod (BMR1 and BMR4) when all hamsters were in standard cages without running wheels (Fig. 3). It seems that seasonal changes in heat loss did not contribute to differences in BMR. In Siberian hamsters the layer of subcutaneous fat decreases in winter (Wade and Bartness 1984), but fur density and its depth increase (Heldmaier et al. 1981a; Paul et al. 2007). It results in constant thermal conductance throughout a year (Heldmaier et al. 1981a; Boratyński et al. 2016). Hamsters had higher BMR at cold than at laboratory temperature and also higher during summer than during winter (Fig. 3), but these differences were independent of seasonal changes in *m*_b_. Our results contradict previous findings that seasonal changes of mass-specific (Heldmaier et al. 1990; Heldmaier and Steinlechner 1981a) but also whole animal BMR are mainly the result of *m*_b_ changes (Heldmaier 1989; Lovegrove 2005; Boratyński et al. 2016). Although BMR is typically negatively related to mean *T*_a_ in the environment and positively correlated with *m*_b_ (Lovegrove 2003; Rezende et al. 2004; Raichlen et al. 2010; White and Kearney 2013; Naya et al. 2018), there is also a considerable intraspecific variation in BMR (Genoud et al. 2018). In Siberian hamsters seasonal changes in BMR may depend on the litter in which hamsters were born or on the degree of seasonal changes in the phenotype (*m*_b_, fur properties, reproductive status, daily torpor; Przybylska-Piech et al. 2021; but see Przybylska et al. 2019a). Variability of seasonal changes in BMR observed in hamsters originating from the same breeding colony (Boratyński et al. 2016; Przybylska et al. 2019a, 2021) but housed under different conditions, highlights the importance of considering and controlling for the cage enrichment and housing conditions during comparative analyses.

One limiting factor to the interpretation of our results is ~ 45% larger floor surface areas in cages with running wheels in comparison to standard cages (528 vs. 363 cm^2^). Individuals in smaller cages might have experienced slightly higher *T*_a_ (Kuhnen 1999), and individuals with access to wheels might have defended slightly lower *T*_b_ than in small barren cages without wheels (Kuhnen 1997, 1999). Therefore, individuals housed in small cages could have lower daily energy expenditure than those in large cages (Steyermark and Mueller 2002). However, although the floor surface area differed between cages, this difference was markedly reduced due to the presence of the wheel. Moreover, in our previous experiments the disparity between cage sizes was more pronounced (~ 0.5 L vs. ~ 17.5 L) and we did not record any differences in BMR, evaporative heat loss, or thermal conductance related to housing conditions, under long or short photoperiod (unpublished data). It supports the conclusion that differences in BMR between individuals housed in cages with wheels and without result from voluntary wheel-running activity.

At moderate temperature (20 °C) hamsters were active for ~ 40% and ~ 60% of the night under short and long photoperiods, respectively. In the cold, long-day hamsters decreased their activity from 60 to 40% of the night, whereas under short photoperiod, nighttime activity was only 8% lower in the cold than at moderate temperature (Fig. 4). This is consistent with previous studies showing that *T*_a_ modifies activity patterns (Tokura and Oishii 1985; Lee et al. 1990; Thomas et al. 1993). Siberian hamsters acclimated to a L12:D12 cycle and kept at 25 °C during the day and 10 °C at night, were more active, and began nighttime activity earlier than animals kept constantly at 25 °C (Tokura and Oishii 1985). Similarly, in ground squirrels (Lee et al. 1990) and mice (Vaanholt et al. 2007) wheel running activity decreased with ambient temperature, suggesting that the heat generated by running did not compensate heat loss in the cold. Conversely, spontaneous cage activity in mice, but not rats, increased during the resting phase of the day when *T*_a_ decreased, probably to generate additional heat (Swoap et al. 2004). Three hypotheses offer explanation for the link between heat derived from activity and heat necessary for thermoregulation. Addition hypothesis proposes that heat generated during exercise may be added to the thermogenesis during rest, substitution hypothesis suggests that heat from exercise may replace heat required for thermoregulation, and partial substitution hypothesis merges the above two, suggesting that first one is valid in warm temperatures while second one in the cold (Wunder 1970; Chappell et al. 2004; Vannholt et al. 2007). Wunder (1970) suggested that heat produced by forced high-speed running partially substituted for cold-induced thermogenesis while at low speed these two sources of heat were additive because of increased thermal conductance caused by increased peripheral blood flow and convection. We did not measure hamsters’ running speed but it seems that disrupted pelage insulation was not a case.

One could expect that BMR should correlate with activity and could be explained by either performance or allocation models (Careau et al. 2008). According to the performance model increased activity leads to increase in BMR to support higher daily energy expenditure. Conversely, according to the allocation model, energy spent on BMR limits the amount of energy available for proactive behaviors (or activity limits energy available for BMR). Both models are supported by experimental results (Careau et al. 2008; Bouwhuis et al. 2014; Gębczyński and Konarzewski 2009). However, we found no correlation between BMR and voluntary activity in the running wheel. Similarly, our previous study on Siberian hamsters showed no correlation between different behavioural traits (including activity in open field) and BMR (Przybylska et al. 2019a). These findings support the independent model, which assumes that BMR is independent of activity (Careau and Garland 2012). Since skeletal muscles used during activity do not contribute to BMR measured in resting animals (Barceló et al. 2017), we should rather expect an increase in total energy expenditure than increase in BMR. Also Kane et al. (2008) found no correlation between voluntary activity and BMR in mice selected for high wheel-running activity. Their BMR also did not differ from BMR of control animals (Kane et al. 2008). However, wheel-running activity prevented an age-related decline in BMR in rats (Ichikawa et al. 2000) or increased RMR (measured at 20–22 °C) in older animals (Goodrick 1980).

Hamsters with access to running wheels increased their *m*_b_ despite short days. When *T*_a_ decreased to 7 °C, *m*_b_ increase was impeded and it was maintained constant, likely reflecting costs of thermoregulation in cold (Fig. 2). Previous data showed that exercising Siberian hamsters had lower fat content in summer than sedentary animals suggesting that voluntary activity prevents seasonal changes (summer increase) in adiposity (Scherbarth et al. 2007). However, the pattern of changes in *m*_b_ due to exercise is not uniform and may be related to seasonality. Mice and rats housed with running wheels decrease their *m*_b_ (Goodrick 1980; Lambert and Noakes 1990; Swallow et al. 1998). In exercising male golden hamsters (*Mesocricetus auratus*) lean and fat mass increased but it did not change when expressed in relation to *m*_b_ (Gattermann et al. 2004). In contrast, fat content in females was reduced (Borer et al. 1983). An increase in *m*_b_ in exercising short day Siberian hamsters was independent of photoperiodically controlled hypothalamic gene expression involved in seasonal *m*_b_ regulation (Petri et al. 2014). It highlights the complexity of pathways underlying seasonal response. It was previously shown that access to running wheels advanced testicular recrudescence (Scherbarth et al. 2007) and inhibited torpor in Siberian hamsters while seasonal moulting was unaffected (Thomas et al.^1993^; Scherbarth et al. 2007, 2008). Access to running wheels also inhibited testicular regression in response to short days in Syrian hamsters, *Mesocricetus auratus* (Gibbs and Petterborg 1986). Similarly, golden mantled ground squirrels (*Citellus* = *Callospermophilus lateralis*) did not enter or delayed entry into hibernation when provided with a running wheel (Pengelley and Fisher 1966). Taken together, present results as well as results of earlier studies clearly call for caution while providing cage enrichment, which may lead to increase in locomotor activity and following aftereffects.

## Effects of nesting material

Availability of nesting material did not affect BMR, which was unexpected as nests can significantly modify thermal environment, and temperatures in the center of the nest may be > 10 °C higher than the surrounding air (Gaskill et al. 2013). Nest building behaviour and nest size were increased in short days and low *T*_a_ (Puchalski et al. 1988; Przybylska et al. 2019b). In brown lemmings (*Lemmus trimucronatus*), a nest provided 46% reduction in thermal conductance, which resulted in 43% lower resting metabolic rate at *T*_*a*_ = − 16 °C comparing to lemmings without the nests (Casey 1981). However, study on BMR and nest building in seven muroid species showed no correlation between presence of a nest and metabolic rate (MR) and suggested that species with high MR depend on nests to a lesser extent than species with lower MR (Hartung and Dewsbury 1979). Even if the nest allowed for decrease in metabolic rate while in the nest, it did not translate to changes in BMR.

The lack of differences in BMR between hamsters housed with and without nesting material can be interpreted in three non-mutually exclusive ways: (1) a barren cage does not induce thermal stress in hamsters kept in cold, (2) hamsters with nests have lower metabolic rate only when they are in a cage while BMR does not differ, or (3) amount of nesting material does not strongly affect physiology and thus should not confound experimental results. Van de Weerd et al. (1997) showed that nest-building material provided to mice (3 per cage) did not affect behavioural or physiological parameters indicative of stress. Furthermore, Bailoo et al. (2018) found that behavioural traits were more sensitive to the environmental conditions than physiological ones and that there was no uniform pattern of the effects of cage enrichment on animal physiology. Thus, since the environmental enrichment of extra nesting material improves animal welfare, we suggest it can be used for studies comparing physiological traits like metabolism without concern.

## Effects of housing in pairs

Siberian hamster is rather solitary than social (Wynne-Edwards 2003) but can be housed in the groups of the same-sex littermates (Jefimow et al. 2011). In such groups winter decrease in *m*_b_ is shallower than in solitary animals, what suggests reduced energy expenditure (Jefimow et al. 2011). In social animals group housing may reduce energy expenditure by reduction in surface-to-volume ratio (Contreras 1984), with the highest energy savings reported for groups of up to five individuals at temperatures below thermoneutrality (Contreras 1984; Canals et al. 1989, 1997, 1998; Gilbert et al. 2010; Canals and Bozinovic 2011). Although pair-housed hamsters were always seen sleeping in close contact, our prediction that the presence of littermates would alleviate cold stress and that pair-housed animals would have lower BMR than animals housed singly was not supported. Single and pair-housed hamsters also did not differ in *m*_b_. Nuñez-Villegas and co-workers (2014) reported that housing in groups decreases BMR in the common degu, *Octodon degus*. Cold-acclimated (*T*_a_ = 15 °C) animals housed in group of three had lower BMR by 15% than animals housed alone. Increasing the group number from three to five resulted in further reduction in BMR by ~ 40%. A reduction in BMR, although smaller (~ 7%), was also recorded in animals acclimated to warm temperatures (30 °C), housed in groups of 3 or 5 individuals (Nuñez-Villegas et al. 2014). Moreover mice kept in groups had less brown adipose tissue (BAT) than mice kept individually, suggesting that social thermoregulation may substitute non-shivering thermogenesis (Heldmaier 1975a). Our results concur with results of Contreras (1984), who reported that huddling in the nest did not affect the metabolic rate of individual laboratory mice and Mongolian gerbils (*Meriones unguiculatus*). It is possible that hamsters housed in pairs benefited from huddling when in cage, but larger group would be necessary to trigger changes in BMR. Two huddling mice (*Mus musculus*) at 12.5 °C reduce their oxygen consumption by ~ 18%, while 3 to 6 individuals huddling together increase energy savings to ~ 30% (Contreras 1984). As an efficiency of huddling depends on the morphological characteristics of the geometric bodies (Canals 1989) and a reduction in metabolic rate results from a reduction in the surface-to-volume ratio, three dimensional huddles may provide larger energy benefits than linear huddles of several or two individuals (McKechnie and Lovegrove 2001; Calf et al. 2002). Thus, it is possible that huddling leads to energy savings when animals are in a huddle, but does not lead to long-term changes in the metabolism of individual animal. Although housing in groups or pairs provides social contact, introducing new animal into cage must be done carefully. Especially in species with clear social hierarchy or in solitary ones. O’Connor and Eikelboom (2000) reported that rats, which were housed singly and then were moved to paired housing showed stress-induced decrease in feeding. Yet, after few days rats can benefit from group housing thanks to social contact or thermoregulation. Group housing, although recommended for animal welfare, may elicit aggressive behaviour. Prevalence of aggression-related injuries in male mice housed in groups is strain–specific and was estimated at ~ 1.5% (Lidster et al. 2019). However, aggressive bahaviours may be reduced by cage enrichment (Ambrose and Morton 2010; Giles et al. 2018). Van Loo et al. (2002) found that male mice housed in groups of three were less aggressive when nesting material was provided to the cage, while the effect of providing wire shelf (the Utrecht Shelter) was opposite. These data indicate that depending on the species, strain, or even ambient temperature, cage enrichment may not fulfill its expected role.

## Conclusion

It seems that there is no simple, general answer to the question of how cage enrichment affects animal behaviour and physiology. As concluded by Van de Weerd et al. (2002), it largely depends on the parameter measured in experimental studies. To avoid inconsistencies among experimental results that could arise from different housing conditions, Sztainberg and Chen (2010) proposed standard cage enrichment for mice. They combined different items, like tubes, wheels, and nest boxes, to cover all animal needs: social, sensory, cognitive, and motor. An enriched environment is known to reduce anxiety in laboratory mice (Ambrose and Morton 2010; Giles et al. 2018), but the same standard items should be applied to other species with caution. Undoubtedly, cage enrichment enhances animal welfare. However, we found that access to a running wheel increased BMR independent of body mass, photoperiod, and ambient temperature. Thus, we suggest not providing running wheels in studies focused on energetics or thermoregulation, especially in animals with distinct seasonal phenotypes.
