# Correction to: Housing conditions modify seasonal changes in basal metabolism and body mass of the Siberian hamster, *Phodopus sungorus*

**DOI:** 10.1007/s00360-022-01440-x

**Published:** 2022-05-06

**Authors:** Małgorzata Jefimow, Anna S. Przybylska-Piech

**Affiliations:** 1 Department of Animal Physiology and Neurobiology, Faculty of Biological and Veterinary Sciences, Nicolaus Copernicus University, ul. Lwowska 1, 87‑100 Toruń, Poland; 2 Department of Vertebrate Zoology and Ecology, Faculty of Biological and Veterinary Sciences, Nicolaus Copernicus University, ul. Lwowska 1, 87‑100 Toruń, Poland

## Correction to: Journal of Comparative Physiology B 10.1007/s00360-022-01434-9

The article “Housing conditions modify seasonal changes in basal metabolism and body mass of the Siberian hamster, Phodopus sungorus”, written by Małgorzata Jefmow, Anna S. Przybylska-Piech was originally published electronically on the publisher’s internet portal (currently SpringerLink) on March 29, 2022 without open access. This article is licensed under a Creative Commons Attribution 4.0 International License, which permits use, sharing, adaptation, distribution and reproduction in any medium or format, as long as you give appropriate credit to the original author(s) and the source, provide a link to the Creative Commons licence, and indicate if changes were made. The images or other third party material in this article are included in the article’s Creative Commons licence, unless indicated otherwise in a credit line to the material. If material is not included in the article’s Creative Commons licence and your intended use is not permitted by statutory regulation or exceeds the permitted use, you will need to obtain permission directly from the copyright holder. To view a copy of this licence, visit http://creativecommons.org/licenses/by/4.0/.

The original article has been corrected.

